# Primary Human Uterine Leiomyoma Cell Culture Quality Control: Some Properties of Myometrial Cells Cultured under Serum Deprivation Conditions in the Presence of Ovarian Steroids

**DOI:** 10.1371/journal.pone.0158578

**Published:** 2016-07-08

**Authors:** Camila Bonazza, Sheila Siqueira Andrade, Joana Tomomi Sumikawa, Fabrício Pereira Batista, Edgar J. Paredes-Gamero, Manoel J. B. C. Girão, Maria Luiza V. Oliva, Rodrigo Aquino Castro

**Affiliations:** 1 Department of Gynecology of Universidade Federal de São Paulo, São Paulo, SP, Brazil; 2 Department of Biochemistry of Universidade Federal de São Paulo, São Paulo, SP, Brazil; Virginia Commonwealth University, UNITED STATES

## Abstract

Cell culture is considered the standard media used in research to emulate the *in vivo* cell environment. Crucial *in vivo* experiments cannot be conducted in humans and depend on *in vitro* methodologies such as cell culture systems. However, some procedures involving the quality control of cells in culture have been gradually neglected by failing to acknowledge that primary cells and cell lines change over time in culture. Thus, we report methods based on our experience for monitoring primary cell culture of human myometrial cells derived from uterine leiomyoma. We standardized the best procedure of tissue dissociation required for the study of multiple genetic marker systems that include species-specific antigens, expression of myofibroblast or myoblast markers, growth curve, serum deprivation, starvation by cell cycle synchronization, culture on collagen coated plates, and 17 β-estradiol (E_2_) and progesterone (P_4_) effects. The results showed that primary myometrial cells from patients with uterine leiomyoma displayed myoblast phenotypes before and after *in vitro* cultivation, and leiomyoma cells differentiated into mature myocyte cells under the appropriate differentiation-inducing conditions (serum deprivation). These cells grew well on collagen coated plates and responded to E_2_ and P_4_, which may drive myometrial and leiomyoma cells to proliferate and adhere into a focal adhesion complex involvement in a paracrine manner. The establishment of these techniques as routine procedures will improve the understanding of the myometrial physiology and pathogenesis of myometrium-derived diseases such as leiomyoma. Mimicking the *in vivo* environment of fibrotic conditions can prevent false results and enhance results that are based on cell culture integrity.

## Introduction

The development of *in vitro* cell culture models has greatly facilitated the ability to study how proliferation, apoptosis, and metabolic processes occur in the cellular machinery [1]. However, data obtained from studies using questionable authenticity of cells in culture lead to questionable significance. The knowledge of the basic biology of human cells, particularly human tumor cells, lags far behind that of rodent cells [2]. Therefore, procedures that involve cell culture require constant and appropriate quality control to avoid inter- and intra-species contamination. Uterine smooth muscle layer cells or myometrium, which constitute the uterus wall, are of our particular interest because they can be affected by uterine fibroids, also known as leiomyomas (benign tumors of the myometrium) [3–5]. We report the usefulness of a primary monolayer culture of myometrial cells from tissue biopsies of women with uterine leiomyoma using classical techniques of cell biology.

Thus, this study established primary cultures of human myometrial cells isolated from uterine leiomyoma tumor biopsies and evaluate and compare the expression of smooth muscle markers (α-smooth muscle actin, calponin, and smoothelin) [4, 6–9], fibroblast markers (vimentin) [4, 10], contractile proteins (connexin 43) [4, 11], an inflammatory gene (cyclo-oxygenase-2 (COX-2)) [4, 12], steroid hormone receptors (estrogen receptor α (ESR1), an estrogen receptor β (ESR2), and a progesterone receptor (PGR)) [3, 13]. In addition, we established growth curves, conditions of co-cultured leiomyoma and myometrium cells, serum deprivation, cell cycle synchronization, culture on collagen surface, and E_2_ and P_4_ effects. These distinct myometrial cell models provide and validate useful tools to investigate mechanisms underlying the process of human uterine leiomyoma [4, 14–17].

## Materials and Methods

### Chemicals and Biochemicals

All chemical reagents were of analytical grade. Deionized and ultra-filtered water from the Milli-Q ultrafiltration system was used. The biochemical assays were conducted using commercially available kits.

### Collection of human specimens/biopsies

Myometrial biopsies were collected from premenopausal women undergoing hysterectomy for leiomyoma at the Urogynecology Unit of the Gynecology Department from the Federal University of São Paulo. Patients were not receiving any hormonal treatment at the time of surgery. Normal myometrium tissue (adjacent to myoma) and without any abnormalities, including adenomyosis or malignancies, were also was collected. Table 1 shows the demographic data from patients. The use of these human specimens was approved by the Institutional Ethics Review Board (CEP0858/10) from the São Paulo Federal University (UNIFESP) and carried out in accordance with the Declaration of Helsinki. A written informed consent was signed by each patient who volunteered to participate before the study start.

**Table 1:** Characteristics of Samples Used in the Study.

Patients with Leiomyoma	
(n = 10 from myoma tissue and n = 10 from adjacent tissue	
**Age** (mean ± SEM)	41±1.5
**Race**	Afro-Americans
• **Cycle number**	•
• **Proliferative phase**	• 10
• **Secretory phase**	• -
**Subtype**	Intramural fibroid

### Tissue isolation and cell culture conditions–Immunophenotype

Myometrial tissue samples obtained from women with uterine leiomyoma during the elective hysterectomy procedure was cut up manually into small pieces of approximately 2 mm^3^ and incubated in Dulbecco’s modified Eagle’s medium without phenol red (Sigma–Aldrich) containing collagenase 1A 1.0 mg/mL (Sigma), 1% antibiotic-antimycotic mixture containing 100 IU/ml penicillin, 100 mg/ml streptomycin, and 5 mg/mL gatifloxacin (Invitrogen), and 10% heat-in-activated fetal bovine serum (FBS) for 45 min at 37°C on a shaker. The digested tissue was subsequently cultured by the explant method in a humidified incubator at 37°C and 5% CO_2_ for three weeks; cell passage was routinely conducted using the Versene solution-EDTA (Gibco, MA, EUA). After this period, non-adherent cells were washed out, and adherent cells were characterized by immunostaining with anti-fibronectin integrin beta-1/CD29 from Cell Signaling Technology (Beverly, MA, USA)) conjugated with FITC-488 from Thermo Fisher Scientific (Waltham, MA, EUA), anti-vimentin Alexa Fluor® 594, and Alexa Fluor® 635 phalloidin antibodies (Thermo Fisher Scientific, MA, EUA) and analyzed through confocal microscopy.

### Monitoring Mycoplasma contamination through PCR

Mycoplasma is a frequent and occult contaminant in cell cultures. It can modify many aspects of cell physiology rendering experiments that are conducted with contaminated cells worthless. This prokaryotic organism can pass through filters used to prevent bacterial and fungal contamination and potentially spread to all cultures in a laboratory. Therefore, the use of selective PCR-based methods is recommended to routinely monitor cell cultures. In this study, gatifloxacin (5mg/mL) was used in tissue samples before culture. Briefly, 1 ml of supernatant of semi-confluent monolayers of myometrial cells in culture was collected and mixed with a PCR master mix (Promega, WI, USA) (antisense primer Myc 5’-GGCGAATGGGTGAGTAACACG-3’, sense primer Myc 5’-CGGATAACGCTTCGCACCTAT-3’), one cycle at 95°C for 5 min; 40 cycles at 94°C for 0.5 min, 55°C for 0.5 min, and 72°C for 1 min; one cycle at 72°C for 10 min, and hold at 4°C until samples are removed from the thermal cycler. Amplified products were evaluated in SYBR stained 2% agarose gel after electrophoresis at 100 V for 1 h (including the 100-bp DNA ladder molecular weight marker). Fluorescent gel bands were visualized using a UV transilluminator (method adapted from [18]).

### 3-(4,5-Dimethylthiazol-2-yl)-2,5-diphenyltetrazolium Bromide (MTT) Cell viability assay

The viability of primary cells in the conditions of serum deprivation, collagen coated plates, and treatment with E_2_ (17β-estradiol) and P_4_ (progesterone) was determined by measuring cell redox activity using the MTT assay in three different conditions. In the first condition, cells were plated at 1 × 10^4^ density on 96-well microplates (Corning Inc.) to start serum deprivation; medium was removed after cell attachment and replaced with DMEN/F12 with FBS restriction (0, 0.1, 0.2, 0.5, 1.0, 2.0, and 10%) for 24 or 48 h at 37°C and 5% CO_2_. In the second condition subsequent to serum deprivation, 5 × 10^3^ cells were plated on 96-well microplates (Corning Inc.) coated with 10 μg of collagen I (purified from rat tail tendon using a method previously described [20]) and left to adhere for 4 h in a humidified atmosphere at 37°C and 5% CO_2_. In the third condition, myometrial cells (1 × 10^4^ per well) were seeded to display 70% confluent monolayers in collagen type I coated plates and cultured in DMEM/F-12 without phenol red and supplemented with specific FBS concentration, 100 IU/ml penicillin, 100 mg/ml streptomycin, and treated with 100 nmol/L E_2_ and 100 nmol/L P_4_ for 16 h. Cells from all three conditions were added with 0.5 mg/ml MTT solution (Sigma) in 10 mM phosphate buffered saline at pH 7.4 (PBS) and incubated for a further period of 2 h at 37°C and 5% CO_2_. After incubation, the medium was removed, and cells were washed. Formazan crystals were dissolved in DMSO and measured spectrophotometrically at 540 nm. Primary cells from the ten-myometrial biopsies were subjected to these three described conditions.

### Cell Growth and co-culturing conditions

Two methods were used to determine the growth rate of human myometrial and leiomyoma cells in culture: i) standard hemacytometer counts of dissociated myometrial and leiomyoma cells; or ii) flow cytometer counts in the Accuri C6 (BD, Bioscience). The population doubling time was calculated from the slope of growth curves at mid-log phase. The total number of population size doubling events in successively transferred cultures was calculated according to [14]. Myometrial cells from leiomyoma adjacent tissue were considered as feeder cells in the co-culturing assay; they were seeded in DMEM medium with the appropriate supplementation of FBS (2%) and co-cultured with leiomyoma cells after the feeder cells reaching 30% confluence. The initial combined concentration of feeder and leiomyoma cells was 3–5 × 10^4^ per well.

### Semi-quantitative RT-PCR

The RNA extraction and semi-quantitative PCR were executed as follows: RNA was extracted with the QIAGEN RNAeasy Plus Micro Kit; quality was evaluated by Agilent 2100 Bioanalyzer (Santa Clara, CA). RNA samples were reverse transcribed and amplified with the QIAGEN QuantiTect Whole Transcriptome Kit into cDNA following the manufacturer’s protocol. PCR amplifications and product quantification were performed using the StepOnePlusTM Real-Time PCR System (Applied Biosystems, Foster, CA, USA). PCR reagents were obtained from QIAGEN. Primers were designed using NCBI Primer-BLAST and synthesized at MGH DNA Core Facility. Primer sequences are listed in Table 2.

**Table 2:** Primes sequences for quantitative PCR (5’->3’).

Name/Symbol	Forward Primer Sequence	Reverse Primer Sequence
**Housekeeping genes:**		
• Glyceraldehyde-phosphatedehydrogenase–*GAPDH*	• AAATTGAGCCCGCAGCCTCCC	• CTCGGCTGGCGACGCAAAAGA
• β2-microglobulin– β*2M*	• CTTATGCACGCTTAACTATCTTAACAA	• TAGGAGGGCTGGCAACTTAG
**Target genes:**		
• Calponin–*CNN1*	• CTGAGAGAGTGGATCGAGGG	• TGATCTTCTTCACGGAGCCT
• Connexin 43 –*GJA1*	• GGAGTTCAATCAATCACTTGGCGT	• ACACCTTCCCTCCAGCAGTT
• Cyclo-oxygenase-type 2 –*COX-2*	• CTGCGCCTTTTCAAGGATGG	• CCCCACAGCAAACCGTAGAT
• Vimentin—*VIM*	• GGCTCAGATTCAGGAACAGCATG	• CCTGTCTCCGGTACTCAGTGGAC
• *Estrogen receptor α (ESR1)*	• ACAAGCGCCAGAGAGATGAT	• CAGATTCATCATGCGGAACC
• *Estrogen receptor* β *(ESR2)*	• GCCTTAATTCTCCTTCCTCC	• TACATCCTTCACACGACCAG
• *Progesterone receptor (PGR)*	• GACTGAGCTGAAGGCAAAGG	• TCCAAGACACTGTCCAGCAG
• *Smoothelin*	• GCTGAGGAGCTGATGACTAT	• TTGAGAAGCTGGAGAAGGAG
• *a-Smooth muscle actin (αSMA)*	• CCTGACTGAGCGTGGCTATT	• GATGAAGGATGGCTGGAACA

### Cell characterization after serum deprivation and calcium mobilization through fluorescence microscopy

After serum deprivation, myometrial and leiomyoma cells from women with leiomyoma were cultured on coverslips previously coated with a thin film of reconstituted rat tail collagen (collagen type I purified from rat tail tendon according to the method previously described [19, 20]). These cells were maintained in 2% and 10% FBS for 16 h after attachment and growth to a confluent monolayer. Cells were subsequently immersed and fixed in 4% paraformaldehyde in PBS for 30 min, washed three times in PBS (15 min each wash), and coverslips were incubated in blocking solution (5% FCS) at room temperature for 1 h; these were subsequently incubated with primary antibodies (1:50) overnight at 4°C. Rabbit mouse anti-integrin β1 (Cell Signaling Technology) and mouse anti-vimentin conjugated with Cy3 (Sigma-Aldrich) were used as primary antibodies. After this incubation, coverslips were washed three times in PBS and incubated for 1 h at room temperature with appropriate fluorescent secondary antibodies conjugated to Alexa Fluor® 488 or Alexa Fluor 594 (Molecular Probes/Invitrogen, Eugene, OR). After the secondary antibody incubation, coverslips were washed three times with PBS and incubated with 4,6-diamidino-2-phenylindole (DAPI) in PBS (300 ng/ml) (Sigma) for 1 h. These cells were washed three times with PBS, mounted on glass slides in Fluoromount-G (Electron Microscopy Sciences, Hatfield, PA), and sealed with nail polish. The negative control for immunostaining was performed in blocking solution without primary antibodies. Coverslips were examined in a Zeiss LSM510 scanning inverted confocal microscope and images were analyzed in the LSM Image Browser 3.2 software (Zeiss, Oberkochen, Germany).

The calcium mobilization of human myometrial and leiomyoma cells (5 x 10^3^) was evaluated in coverslip cultured cells (12 mm diameter) incubated with 4 μM Fluo-4/AM (Molecular probes, Invitrogen, USA) and maintained in Tyrode solution in a humidified atmosphere at 37°C and 5% CO_2_. Briefly, cells were stimulated early with KCl (80 mM) and ionomycin (1.0 μM); Fluo-4/AM was excited with an argon laser (λEx = 488 nm) and light emission was detected using a Zeiss META detector (λEm = 500−550 nm). The pinhole device was not used. Images were collected at approximately 5 s intervals. All images were captured and processed using an LSM 510 META confocal microscope (Zeiss, LSM 780, Germany) with a 63 × objective (Plan-Neofluar, 1.4 numerical aperture) under oil immersion. Fluorescence intensity was normalized to basal fluorescence using the Examiner 3.2 (Zeiss, Germany) and Image J (NIH, USA) software.

### Cell cycle evaluation by flow cytometry

Myometrial and leiomyoma cells (5 x 10^5^) were cultured in 10% and 2% FBS (serum deprived) for 36 h in 6-well microtiter plates after reaching sub-confluency, i.e., 50 to 70% confluence and subsequently treated with E_2_ and P_4_ for 16 h. After treatment, cells were harvested and washed with cold PBS. Cells were fixed with 2% paraformaldehyde, permeabilized with 0.01% saponin, and incubated with RNase (4 mg/mL) for 30 min; 5 μl of PI (1 mg/ml) was added after this incubation. The cell-cycle analysis was performed by flow cytometry in the Accuri C6 (BD, Bioscience).

### Protein preparation and Immunoblot analysis—Western blot analysis

At the termination of 2% FBS cultures treated with E_2_ and P_4_ for 16 h, cells were washed twice with PBS and harvested on ice by scraping the dishes with a disposable cell scraper in the presence of lysis buffer consisting of 50 mmol/l Tris–HCl (pH 8.0), 150 mmol/l NaCl, 0.1% sodium dodecyl-sulfate (SDS), 100 mg/ml phenylmethylsulfonyl fluoride (PMSF), 1 mg/ml aprotinin, 1% NP-40, and 0.5% deoxycholic acid (sodium salt) at pH 7.5 containing the Roche Complete Protease Inhibitor Cocktail (Basel, Switzerland), phosphatase inhibitors, 1 mM Na_3_VO_4_ (Sodium orthovanadate), and 100 mM NaF (sodium fluoride); these cells were frozen at -80°C. The total protein content was measured using the Micro BCA Protein Assay kit from Pierce (Rockford, IL, USA). Each 100 mg aliquot of protein extracts was electrophoresed on a 10% and 12% SDS-polyacrylamide gel (SDS-PAGE) and transferred to polyvinylidene difluoride (PVDF) membranes. Membranes were blocked with 5% BSA in Trizma base saline buffer (TBS) at 4°C overnight and incubated at room temperature for 2 h with the anti-Src rabbit and anti-phospho-Src rabbit corresponding primary antibodies (Tyr-416), and anti-FAK rabbit, anti-phospho-FAK rabbit (Tyr-397), anti-ERK 1/2 MAPK rabbit, anti-p130cas rabbit (Y165), anti-p130cas rabbit (Y410), anti-Akt rabbit, anti-phospho-Akt rabbit, anti-pRb rabbit, anti-p21 rabbit, anti-p53 rabbit, anti-p27 rabbit, anti-Bax rabbit, anti-BCl-xL rabbit, and anti-β-actin rabbit antibodies. All antibodies were from Cell Signaling Technology (Beverly, MA, USA) and diluted in 1% BSA in TBST at 1:1000 in TBS. Membranes were further incubated for one hour at room temperature with the appropriate secondary antibodies conjugated with horseradish peroxidase (anti-rabbit IgG, HRP linked antibody, Cell Signaling Technology) diluted in 1% BSA in TBST. After each step, membranes were sequentially washed three times with TBST. Antigen–antibody complexes were detected with the secondary antibody using the ECL chemiluminescence detection system (Amersham, Arlington Heights, IL, USA). The densitometry analysis was performed in the ImageJ software (NIH, USA) using the phospho-proteins/total-proteins ratio and β-actin as controls. These experiments were performed with cultured cells from all specimens collected from patients.

### Statistical analysis

The results are presented as means of three independent experiments. The statistical analysis was performed using GraphPad PRISM5.0 (La Jolla, CA). Briefly, the Student’s t-test was used to compare means between two independent groups whereas one-way ANOVA followed by the Tukey’s post-test was used to compare means between two or more independent groups. Two-way ANOVA was used to compare group means influenced by two independent factors. The error bars represent the SEM in some of the figures and SD in others. The level of p≤0.05 was accepted as significant.

## Results and Discussion

### Transfer of human myometrial and leiomyoma cells in monolayer cultures

**Fig 1:**
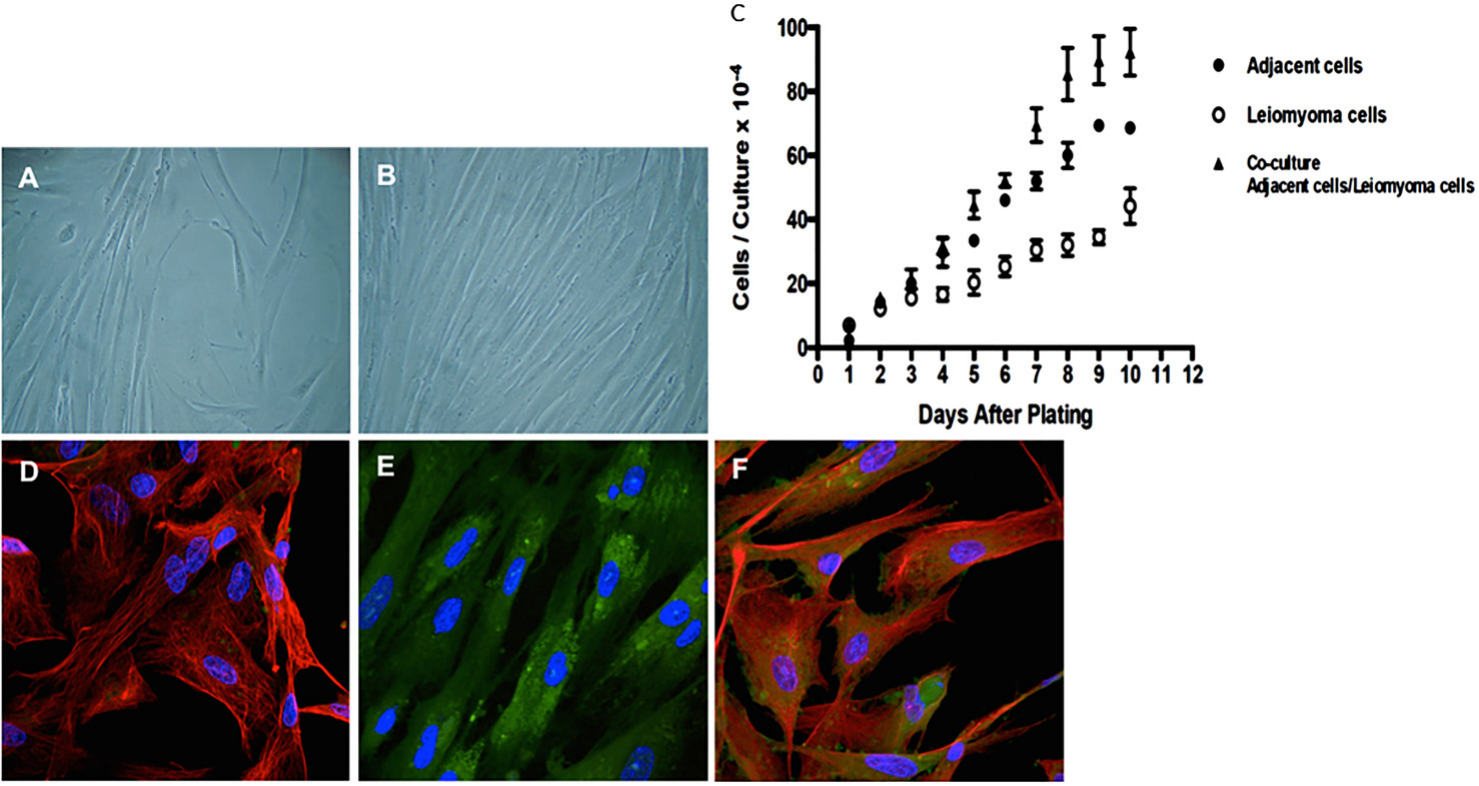
Immunophenotype of leiomyoma and myometrial cells from women with myoma uterine. Adhered and spread cells after tissue disaggregation in collagenase following with primary explants. Cells are shown under phase contrast microscopy and indirect immunofluorescence for phalloidin, fibronectin-integrin β1/CD29, vimentin, and DAPI (blue, for nuclei). (A-B) Phase contrast in the confluent culture of leiomyoma cells after three days. (A) Low density (B) high density (magnification, ×400). Mycoplasma contamination was not observed in any of the processed tissues. (C) Log-phase growth rate by cell counting–growth characteristics of leiomyoma cells, myometrial adjacent cells, and co-cultured myometrial adjacent (as feeders) with leiomyoma cells (on plastic surface). (D-F) Analysis of myometrial markers by confocal microscopy; vimentin and fibronectin integrin β1/CD29. (D) Cytoskeletal organization (Phalloidin, Alexa-594-red); (E) integrin β1/CD29 (FITC-488, green); and (F) Co-localization integrin β1(FITC-488, green) and vimentin (Alexa-594-red). Bar, 10 μm.

The initial stage of cell growth of human leiomyoma and myometrial cells isolated from leiomyoma tumors and respective adjacent myometrium tissue in culture was termed passage 1 (p1). At approximately 14–17 days from the initial isolation and 70–90% confluence (Fig 1A–1C), cells were sub-cultured (passaged) and plated at a density of 3.0 × 10^4^ cells/mL into a 150 cm^2^ culture flask and termed passage 2 (p2). Passage 1 for myometrial cells from tumor-adjacent tissue was established approximately at 9–12 days (mean±SEM) from the initial isolation because they grow faster than cells isolated from leiomyoma tumors (Fig 1C). The co-culture of myometrial cells from tumor-adjacent tissue (as the feeder layer) and leiomyoma cells shows that feeder cells transmit paracrine signals to leiomyoma cells, which show enhanced growth (Fig 1C). Mycoplasma contamination was not observed in any of the processed tissues.

A partial characterization of the primary cells was performed on the third passage; these cells presented a phenotype very similar to that of cells in the original tissues (from uterine leiomyoma). The results showed cells with elongated, spindle-shape morphology, and large size and long cell protrusions in both poles when stained by phalloidin-red and evident presence of integrin-β1 and fibroblast marker vimentin-positive cells by confocal microscopy (Fig 1D–1F). This characterization shows evidence that cultured human leiomyoma and myometrial cells present an intermediate myoblast phenotype or a probable mixture of subset populations of myometrial cells (smooth muscle cells) and myofibroblasts cells [5]. Both myoblasts (undifferentiated myocyte precursors) and myofibroblasts (a type of fibroblast normally involved in wound healing and inflammation, and often defined by the expression of α-smooth muscle actin, which, together with myosin, imparts to contractile powers) express the vimentin-intermediate filament protein [21].

Thus, the conventional pathological definition describing fibroids as clonally derived from the proliferation of a single clone of smooth muscle cells do not represent the cell populations present in the tumor. The cellular heterogeneity of fibroids [22] and the fact that myoblasts, myofibroblasts, or fibroblasts constitute major cellular components of fibroids that can be recognized by routine IHC and cell sorting are dismissed or ignored in some studies [23–25].

### MTT assay–serum deprivation curve

**Fig 2:**
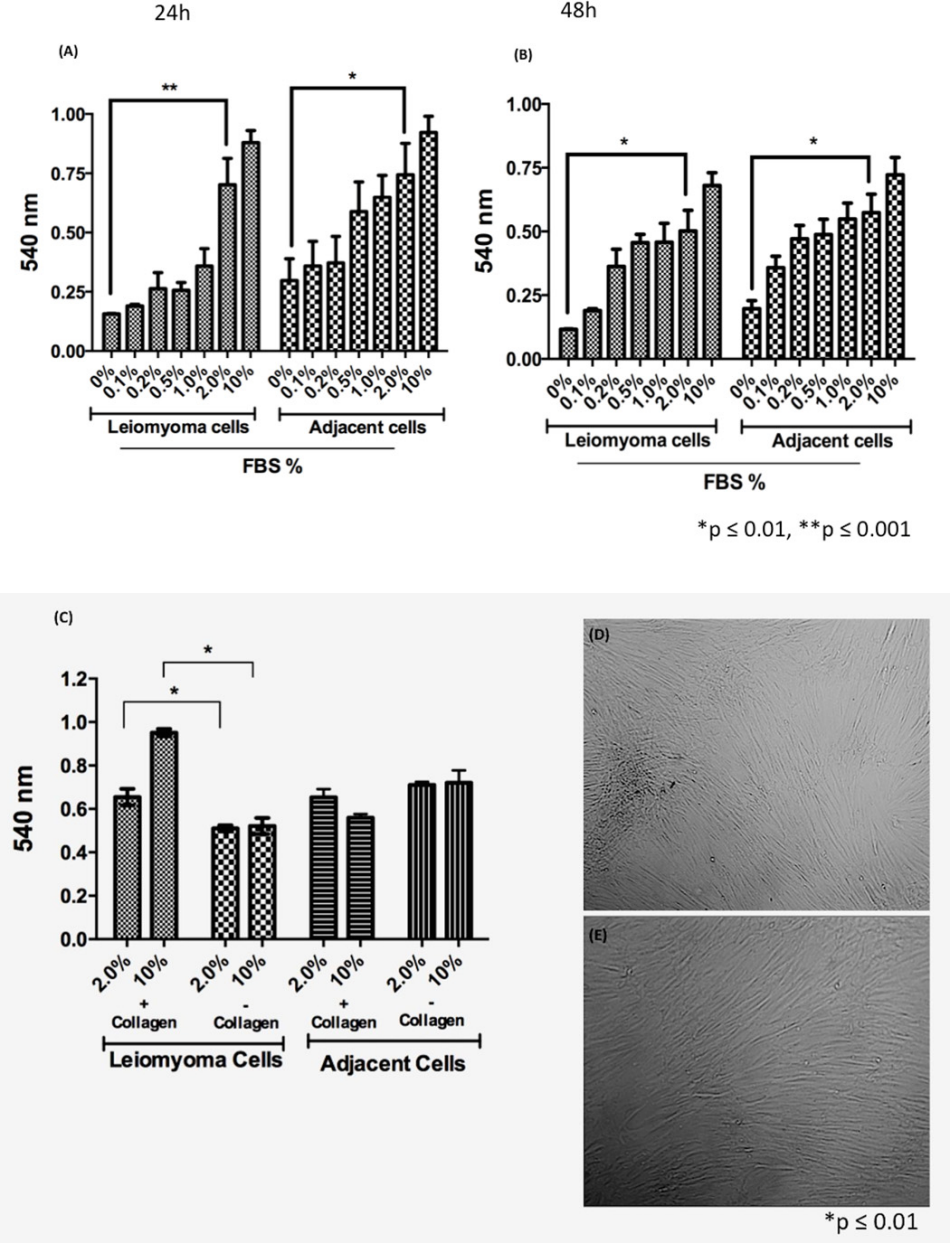
Cell viability by the MTT assay. (A and B) Leiomyoma and myometrial adjacent cells (1 × 10^4^) were maintained in serum deprivation with different concentrations of FBS (0%-10%) for 24 and 48 h in 96-well microtiter plates. (C) Cell viability of uterine leiomyoma cells and myometrial adjacent cells cultured in two concentrations of FBS (2% and 10%); cells (5 × 10^3^) were seed on plastic and on a collagen type I coated plates. (D and E) Phase contrast of confluent culture of leiomyoma cells and myometrial adjacent cells on collagen type I coated plates. The morphology of leiomyoma cells is not altered; these cells were less spread than the myometrial adjacent cells when seeded onto collagen type I-coated plates. Mycoplasma contamination was not observed in any of the processed tissues.

The screening of growth response under conditions of serum deprivation was performed on leiomyoma and myometrial cells using medium with different concentrations of FBS (0, 0.1, 0.2, 0.5, 1.0, 2.0, and 10%). Growth was monitored by phase-contrast microscopy and the MTT assay. The MTT assay results showed significant growth in 2% and 10% FBS medium based on the ability of dehydrogenases of viable cells to reduce the MTT reagent to formazan at 24 and 48 h (Fig 2A and 2B). The conditions of serum deprivation at the lowest concentration of FBS (1%) decreased the viability of leiomyoma and myometrium cells at 24 and 48 h. Both leiomyoma and myometrial cells remained viable in 2% FBS medium. It is important to note that, high concentrations of serum (10%) inhibit the growth of many types of normal and neoplastic cells in culture; these cells rarely experience high concentrations of serum-associated factors *in vivo*, except acutely after wounding. Some tumor cells are selected *ex vivo* due to their ability to proliferate without an intimate contact with stroma; different cell types do not proliferate in culture because the microenvironment of tumors cannot be reproduced *in vitro* [26]. Thus, human cells isolated from tumor biopsies and propagated under standard tissue culture conditions are induced to proliferate in an environment that is different from that of their original tissues in tumors of cancer patients [27]. The use of cell culture as a model for disease conditions needs to be carefully evaluated.

We successfully cultivated leiomyoma and myometrial cells *in vitro* in collagen type I coated plates as the growth substratum to minimize the lack of interactions because a collagen-rich extracellular matrix (ECM) is observed in leiomyomas. Under these conditions, leiomyoma cells were metabolically viable and showed a growth rate of 25% of that of myometrial cells (Fig 2C). Previous studies [14, 28] show that tumor cells cultured on conventional tissue culture plastic plates grow slower than normal cells derived from the microenvironment of human tumors (Fig 2C).

Under the culture conditions used, most of the leiomyoma and myometrial cells attached to the collagen-coated plates remained viable in 2% FBS medium, did not lose adhesiveness, clearly exhibited elongated, spindle-shape, and polygonal morphology that are typical of myofibroblasts, and proliferated more than cells cultivated in the other studied conditions (Fig 2D and 2E).

### RT-PCR analysis of markers of smooth muscle cells and fibroblasts

**Fig 3:**
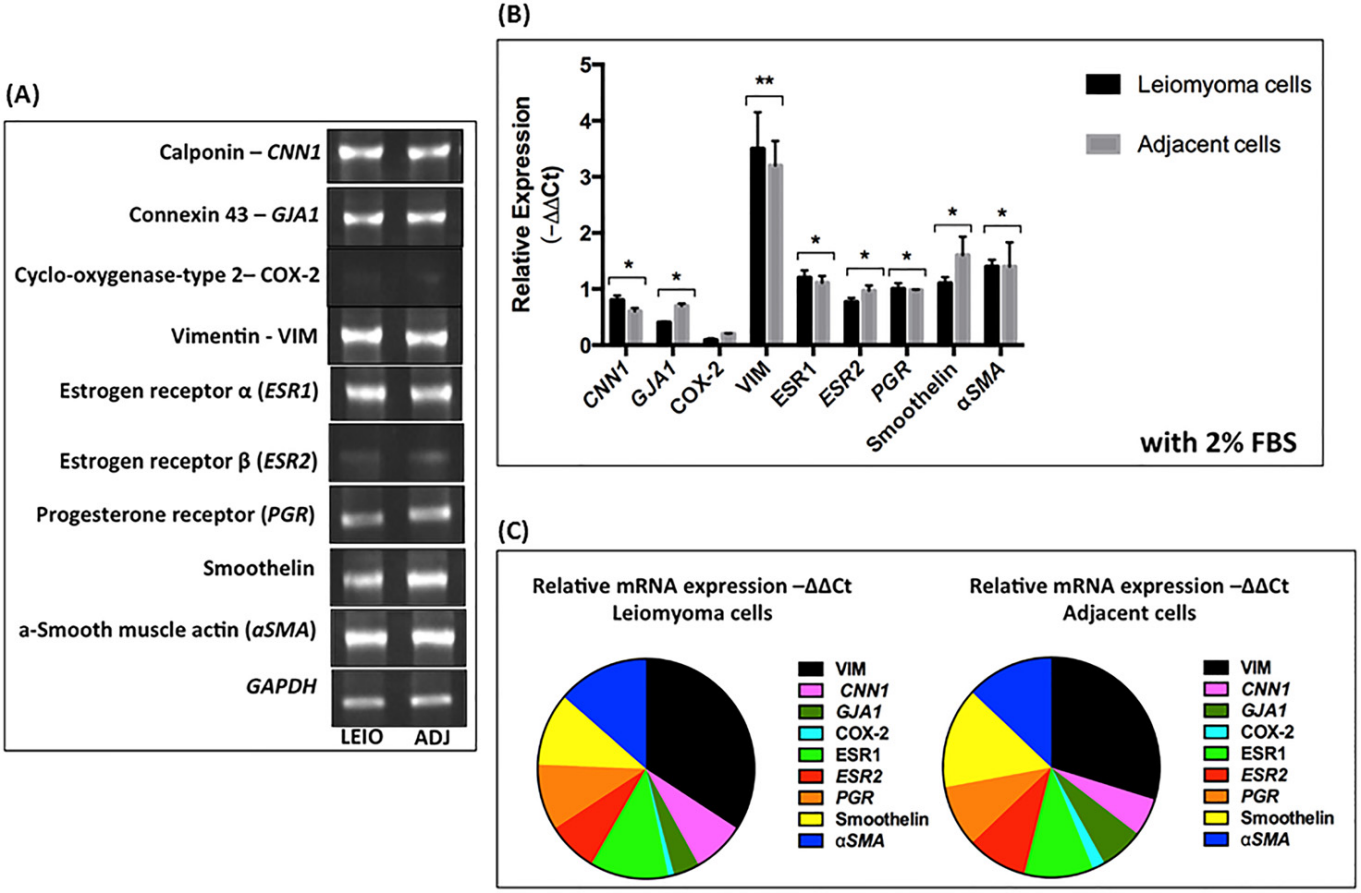
mRNA expression of smooth muscle cell markers and ovarian steroid receptors in leiomyoma and myometrial adjacent cells. (A) Representative agarose gel demonstrating the expression of nine genes in leiomyoma and myometrial adjacent cells. (B) Relative expression (-ΔΔCt) of the nine genes. *P≤ 0.01, **p ≤ 0.001 by the Student’s t-test, samples showed significant changes in gene expression; no significant changes in gene expression was observed in all other conditions. (C) Parts of the whole. Each row defines the same result of (B) with the relative mRNA expression of nine genes.

The expression levels of human myometrium-associated genes were evaluated in leiomyoma and myometrial cells through RT-PCR on mRNA from leiomyoma and myometrial cells cultured in 2% FBS and collagen coated plates. The results demonstrated that the mRNA expression of vimentin (VIM, a myoblast and myofibroblast marker), calponin (CNN1, a smooth muscle marker), α-smooth muscle actin (αSMA), estrogen receptor α (ESR1), smoothelin (smooth muscle markers), and progesterone receptor (PGP) were significantly higher than the expression of estrogen receptor β (ESR2), connexin 43 (GJA1, a gap junction protein), and cyclo-oxygenase-type 2 (COX-2) in both tissues (Fig 3A–3C). Interestingly a difference between the expression patterns of leiomyoma and myometrial cells was observed; leiomyoma cells showed more prominent expression of *VIM*, *ESR1*, CNN1, and *αSMA* than myometrial cells, and myometrial cells showed more prominent expression of *GJA1*, *smoothelin*, and COX-2 than leiomyoma cells (Fig 3A–3C). These results represent the second evidence that cells extracted from leiomyoma and myometrium adjacent tissue represent a mixed population of myometrial smooth muscle cells and myoblast cells. The estrogen receptor-β (ESR2) was detected in both leiomyoma and adjacent myometrium, which is consistent with the relatively low ESR2 expression reported in non-pregnant human myometrium [4, 5, 29].

**Fig 4:**
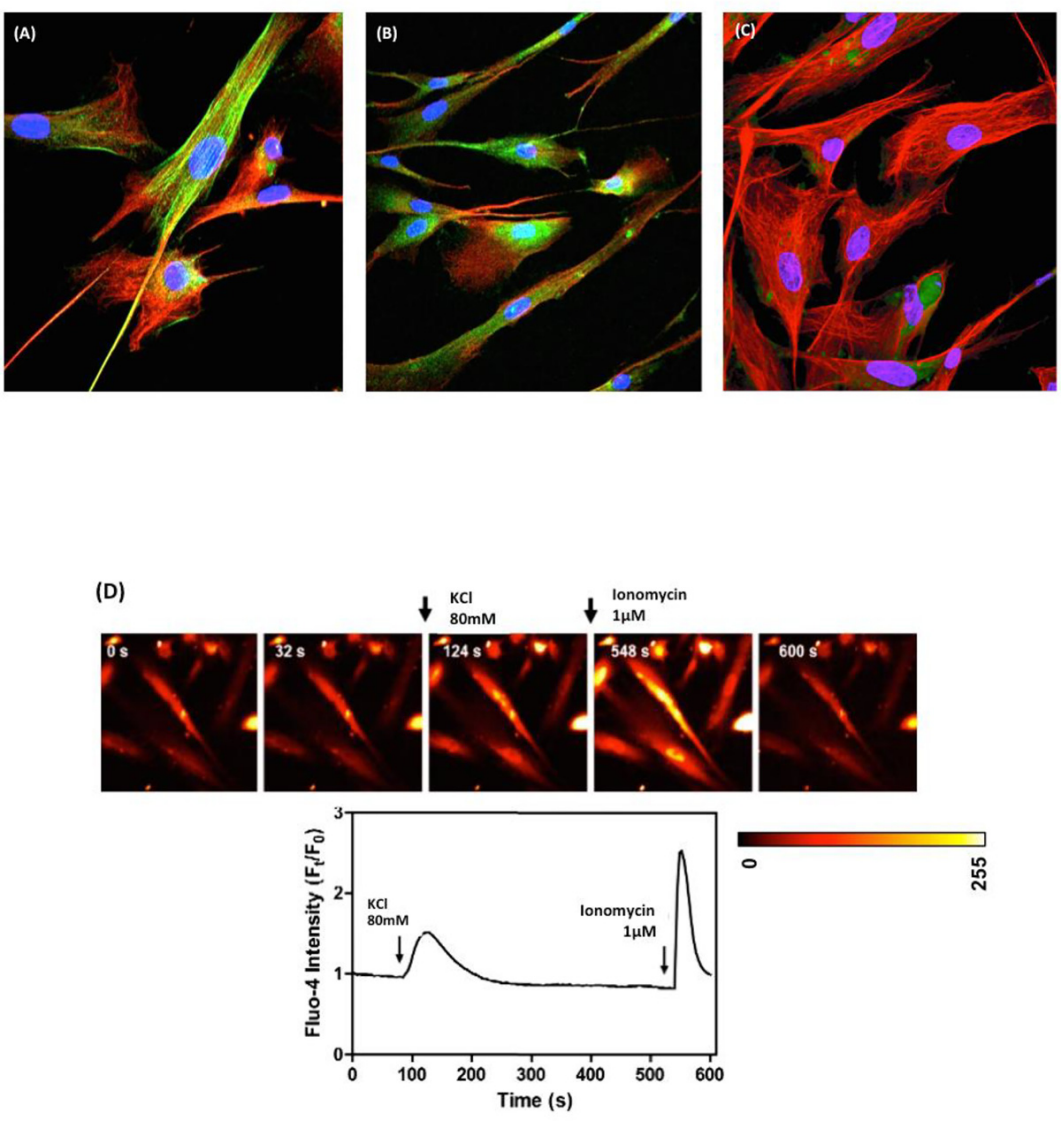
Differentiation of leiomyoma and myometrial adjacent cells in conditions of serum deprivation. The concentration of FBS in the medium was changed from 10 to 2% when the leiomyoma cells reached 30 to 60% confluence. We analyzed the myofibroblast markers vimentin-green, CD29 (FITC-488), and cytoskeleton with phalloidin-red. (A) Cytoskeletal organization (Phalloidin, Alexa-594-red) and integrin β1/CD29 (FITC-488, green); (B) Co-localization of integrin β1/CD29 (FITC-488, green) and vimentin (Alexa-594-red); an initial fusion of the muscle fibers. (C) Bar, 10 μm. (C) Cytoskeletal organization (Phalloidin, Alexa-594-red) and integrin β1/CD29 (FITC-488, green) showing loss of CD29 expression. (D) The intracellular concentration of Ca^2+^ is increased in leiomyoma cells induced by KCl (80 mM). Leiomyoma cells loaded with Fluo-4AM for 40 min were stimulated with 80 mM KCl (at time 100 sec), and the intracellular Ca^2+^ was measured by laser confocal scanning microscopy. Representative pseudo-colored images are shown concerning the basal intensity on a fluorescence intensity scale ranging from 0 (black or blue) to 255 (white or red). All content of Ca^2+^ was mobilized by ionomycin (1 μM).

The complete characterization of leiomyoma and myometrial adjacent cells was based on the morphological analysis. The confocal image using the same previously studied markers (phalloidin (cytoskeleton), vimentin (myoblast and fibroblast marker), and integrin β1/CD29 (myoblast marker) showed the initial fusion of muscle fibers (Fig 4A–4C). Because myometrial cells extracted from both tissues were first obtained with 10% FBS, we hypothesized that changes in these cells in 2% FBS may have led myoblasts to differentiate into myocytes. In addition, these cells may represent a mixed population of myometrial smooth muscle cells and myoblasts; however, one cell type may be predominant in this mixture. To verify this hypothesis, we investigated the Ca^2+^ mobilization induced by KCl (80 mM). We observed that Ca^2+^ mobilization was triggered by KCl depolarizations with one Ca^2+^ peak and the average amplitude of Ft/F0 1.8 ± 0.5 (Fig 4D). Following the KCl depolarizations, the total Ca^2+^ content was released using 1 μm ionomycin in 250 nm external Ca^2+^ medium. These results clearly show that cultured human leiomyoma cells from patients with leiomyoma may represent an intermediate phenotype between that of myoblasts and myocytes. Previous studies [25] identified four subpopulations of cells in fibroid tissue, which also confirms the expression of markers of smooth muscle cells and myoblasts. Cells from adjacent myometrium tissue show a slight difference from leiomyoma cells with a prominent expression of smooth muscle markers (previously shown in Fig 3A–3C).

### Estrogen and progesterone effects in cultured human leiomyoma and myometrial cells–Mitogen-activated protein kinases (MAPK) signaling

**Fig 5:**
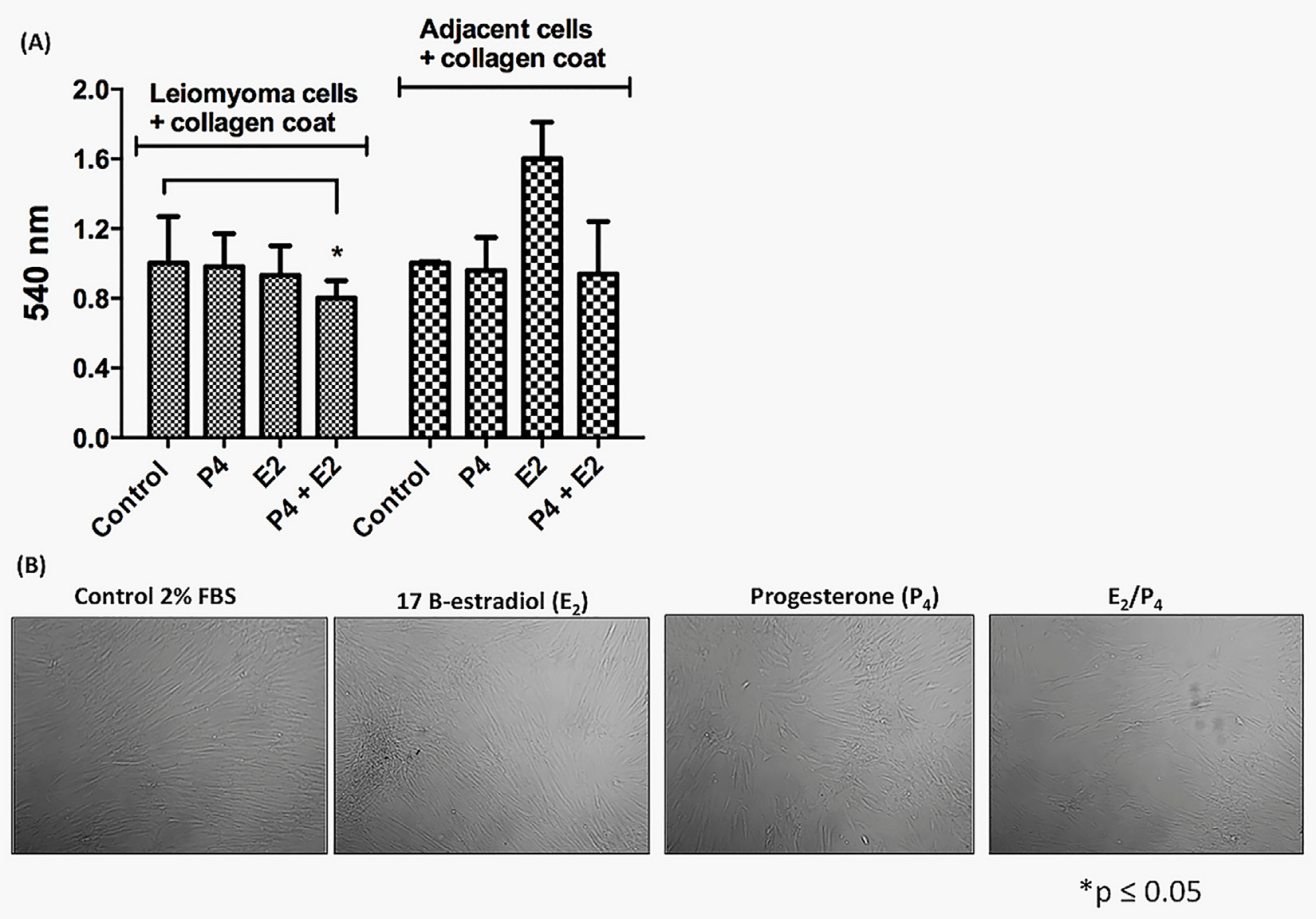
Effect of steroid hormones (E_2_ and P_4_) on the viability of leiomyoma and myometrial adjacent cells. (A) Leiomyoma cells (5 × 10^3^) and myometrial adjacent cells were treated with E_2_ (100 nmol/L) and P4 (100 nmol/L) for 24 h in 96-well microtiter plates. Cell viability was assessed by the MTT reduction test. (B) Phase contrast–confluent culture of leiomyoma and myometrial adjacent cells after treat with E_2_ (100 nmol/L) and P_4_ (100 nmol/L). The statistical significance was evaluated using one-way ANOVA followed by the Tukey's test. A p-value of ≤0.05 was considered to indicate significance (*). These experiments were performed with cultured primary cells from specimens collected from patients.

**Fig 6:**
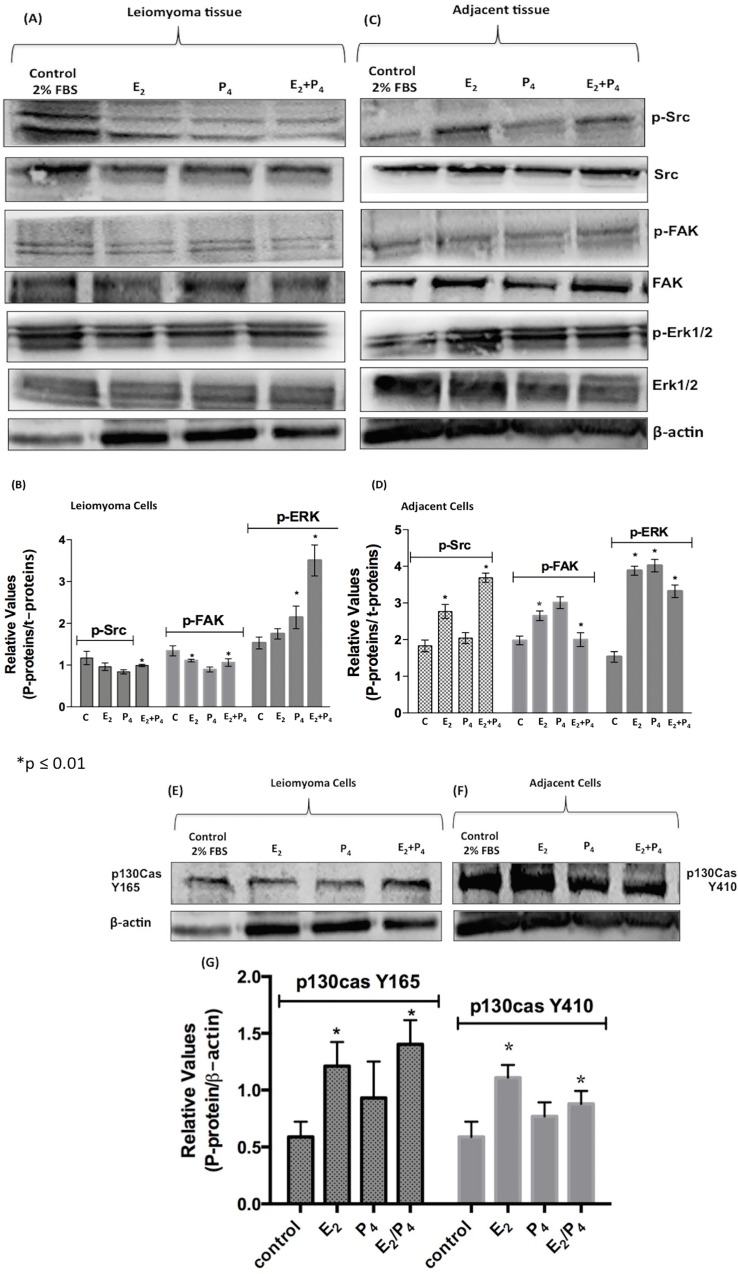
Detection of signaling phosphoproteins by Immunoblot analysis in leiomyoma and myometrial adjacent cells. Leiomyoma and myometrial adjacent cells (5 x 10^5^) were treated with E_2_ (100 nmol/L) and P_4_ (100 nmol/L) for 16 h in 6-well microtiter plates; lysate proteins were separated by 10% SDS-PAGE and electro-transferred to nitrocellulose membranes. Membranes were blocked and incubated with rabbit primary antibodies, (A and C) anti-phospho-Src (Tyr-416), anti-Src, anti-phospho-FAK (Tyr-397), anti-FAK, anti- phospho-Erk1/2 MAPK, anti-Erk1/2 MAPK, (E) anti-p130Cas Y165, (F) anti-p130Cas Y410, (H and J) anti-phospho-Akt, anti-Akt, and anti-β-actin. (B, D, G, I, and L) Graph bars represent the densitometric analyses of the immunoblotting results. The results are represented as band intensities in arbitrary units relative to the respective total phopho-proteins load and total control (β-actin) load. Antibody binding was visualized by chemiluminescence, and the relative levels of these proteins were determined by the densitometric analyses. These experiments were performed with cultured primary cells from specimens collected from patients (*p<0.01).

A salient common feature of leiomyomas is their responsiveness to steroid hormones, thus providing an opportunity for intervention [30]. Therefore, we investigated the response of leiomyoma and myometrial cells to the steroid hormones, E_2_ and P_4_, tested individually and in combination. At first, the MTT cell viability assay was used to evaluate the effect of E_2_ (100 nmol/L) and P_4_ (100 nmol/L) on both leiomyoma and myometrial cells. E_2_ slightly decreased the viability of primary leiomyoma cells (Fig 5A). However, the E_2_+P_4_ hormone combination clearly decreased the viability of leiomyoma cells after 24 h of treatment (Fig 5A). In addition, we observed a change in leiomyoma cells morphology after hormone exposure (Fig 5B). These viability alterations in leiomyoma cells result from the genomic and cytosolic effects of steroid hormones that encompass activation of mitogen-activated protein kinases, adenylyl cyclase, protein kinase C and A, and heterotrimeric guanosine triphosphate-binding proteins (G-proteins) [13, 31]. The involvement of the Src pathway was studied to gain insight into the steroid hormone signaling involved in primary leiomyoma cells. The results showed that the treatment of leiomyoma cells with the hormone combination (E_2_ and P_4_ (100 nmol/L)) led to a significant Src-Tyr-416 activation and light FAK-Tyr-397 activation with pSrc/total Src and pFAK/total FAK ratios of 0.94 ± 0.1 and 1.2 ± 0.4, respectively (Fig 6A and 6B). In the myometrial cells, the exposure to E_2_ (100 nmol/L) and hormone combination (E_2_ + P_4_) (100 nmol/L) induced Src-Tyr-416 activation and slightly induced FAK-Tyr-397 phosphorylation (Fig 6C and 6D). As expected, the treatment with steroid hormones resulted in increased ERK1/2 phosphorylation mainly in the leiomyoma cells exposed to the hormone combination (E_2_+P_4_) with a pERK1/2/total ERK1/2 ratio of 3.6 ± 0.2 (p≤0.01 and p≤0.001, Fig 6A and 6B respectively). These results confirm that cultured primary leiomyoma cells are responsive to steroid hormones. Moreover, we observed that focal adhesion sites are p130cas dependent in these leiomyoma cells. Unprecedentedly, we observed the recruitment of tyrosine-165 (Tyr165) phosphorylation residue in leiomyoma cells, and Tyr410 phosphorylation in adjacent myometrial cells (Fig 6E–6G). The difference between Tyr residue recruitment and phosphorylation on p130cas requires further investigation. These observations may indicate that the Src–p130Cas complex can be more effective at increasing adhesion than the Src–FAK complex in these cultured conditions [32]. Finally, the participation of the extranuclear phosphatidylinositol 3-kinase-AKT pathway in leiomyoma cells was investigated. A slight phosphorylation of AKT was required under these conditions when human leiomyoma cells were stimulated with the combination of steroid hormones (Fig 6H and 6I). AKT phosphorylation was observed in myometrial adjacent cells in the presence of P_4_ and the combination of steroid hormones (3.4 ± 0.4, and 5.2 ± 1.2, p≤0.01, respectively, Fig 6J and 6L). These results are in agreement with those reported by others authors describing P4 as the AKt activator pathway in fibroid cells promoting cell survival [3, 33].

### Cell-Cycle Synchronization and Effects of E_2_ and P_4_ on Leiomyoma and Myometrial cells

**Fig 7:**
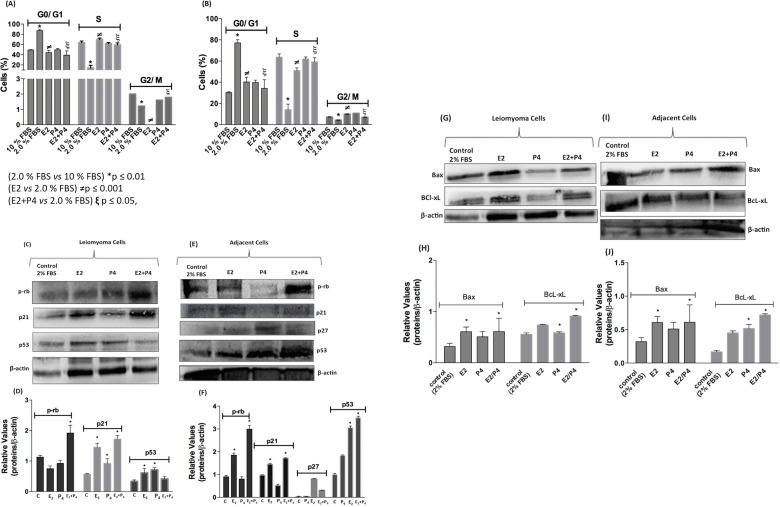
Cell-Cycle Synchronization and Immunoblot analysis of cell-cycle and cell death proteins. (A and B) Percentages of populations of leiomyoma and myometrial cells (1 × 10^5^). Each cell cycle phase was evaluated by flow cytometry using propidium iodide (PI) staining after treatment with E_2_ (100 nmol/L) and P_4_ (100 nmol/L) for 16 h. The statistical significance was evaluated using one-way ANOVA followed by the Tukey's test. (2.0% FBS vs 10% FBS) *p ≤ 0.01; (E_2_ vs 2.0% FBS) ≠p ≤ 0.001; (E_2_+P_4_ vs 2.0% FBS) ξp≤0.05. These experiments were performed with cultured primary cells from specimens collected from patients. (C and E) Effect of E_2_ (100 nmol/L) and P_4_ (100 nmol/L) on the expression of the cell cycle regulatory proteins pRb, p21, p27, and p53 in both types of leiomyoma and myometrial cells (5 × 10^5^) for 16 h. Lysate proteins were separated by 12% SDS-PAGE and transferred to PVDF membranes. Membranes were incubated with anti-pRb, anti-p21, anti-p27, anti-p53, and anti-β actin (control) antibodies; antibody binding was visualized by chemiluminescence. (G and I) Effect of E_2_ (100 nmol/L) and P_4_ (100 nmol/L) on the expression of the cell death proteins Bax (pro-apoptotic) and BcL-xL (anti-apoptotic) in both types of myometrial cells (5 × 10^5^) for 16 h using the same assay conditions. (D, F, H, and J) The relative levels of proteins were determined by the densitometric analyses using the ImageJ (San Diego, CA, USA) imaging software with β-actin as the control for each sample. These experiments were performed with cultured primary cells from specimens collected from patients. Values are expressed as means ± standard deviation.

The transition between G0 quiescence and early G1 is regulated by growth-stimulatory and growth-inhibitory factors present in the extracellular environment. The cell-cycle exit into G0 quiescence can thus be achieved by removing serum from the culture medium because it contains mitogenic factors. Cell-cycle synchronicity is achieved following the addition of serum back into the medium to stimulate cell-cycle entry into the early G1 phase. However, the effectiveness of this method relies on the susceptibility of cells to exit cell cycle into G0 quiescence following serum withdrawal [16, 34]. The understanding of the molecular and biochemical basis of cellular growth, viability, proliferation, gene expression, and translational and post-translational modifications involves the investigation of regulatory events that most often occur in a cell-cycle phase-dependent fashion [16, 35]. The adverse cellular disruptions that occur in cell biology assays invariably require cell cycle induced synchronization. As mentioned, primary cells isolated from biopsies rarely experience large concentrations of serum-associated factors *in vivo*, except acutely after wounding [26]. In this study, leiomyoma and myometrial cells were seeded at sub-confluent conditions in high serum-containing medium (10% FBS) to induce G0 arrest. Cells are washed multiple times with PBS after 18 h, and 2% FBS medium was added back to the cultures. Subsequently, cells entering the S phase were monitored by propidium iodide staining for DNA content and flow cytometer analysis to determine the percentage of cells exiting quiescence and progressing through the cell cycle. We observed that 90% of leiomyoma cells were detected in the G0/G1 cell-cycle phase, in 2% FBS supplementation. A decrease around 50% in the cell population synchronization was observed when steroid hormones (P_4_ (100 nmol/L) and combined E_2_ and P_4_ (100 nmol/L)) were added to leiomyoma cells (Fig 7A). However, only 80% of the population of myometrial adjacent cells was observed in the G0/G1 phase and 60% continued to progress through the cell-cycle into the late G1 phase and S phase in the presence of steroid hormones (combined E_2_ and P_4_ at 100 nmol/L) (Fig 7B). These results may indicate that differential rates of cell cycle progression within the cell population continue to increase, and thereby, lead to unsynchronized cells.

This is an important point to be taken into consideration to ensure the quality of assays and production of reliable results when analyzing and interpreting results from procedures using serum deprivation conditions in cell culture.

The cell cycle process, particularly the transition from one phase to another, is regulated by the activities of different cyclin-dependent kinases (CdKs) and their specific regulatory subunits, which are called cyclins. Each complex CdK/cyclin phosphorylates proteins that are required for cell cycle progression. The catalytic activity of this complex is regulated by binding CdK inhibitors [34]. CdK inhibitors (p21 and p27), p53 (tumor suppressor), and pRb (critical regulator of DNA replication) and their associations with the detection of cell death protein were investigated in the same cell culture conditions. The Western blot analysis showed that the treatment of leiomyoma cells with E_2_ (100 nmol/L) and hormone combination (E_2_ and P_4_ at 100 nmol/L) (Fig 7C and 7D) led to an increased expression of pRb associated with control of myoblast to myocytes differentiation, association between p21 as AKt substrate and cell proliferation and survival, and association between p53 and cell death by apoptosis. Increased pRb expression was observed in myometrial adjacent cells treated with the steroid hormone combination; moreover, p53 increased expression was detected in the presence of E_2_ (100 nmol/L) and its combination with P_4_ (100 nmol/L) (Fig 7E and 7F). The expression of p21 and p27 were detected as light in all treatments. These results were consistent with those observed in Akt phosphorylation and the fact that in part, the compensation of extracellular signals influences the cell cycle machinery through their ability to control the levels of CDK inhibitors and their intracellular localization. During the G1 phase, growth factors induced the p21 expression on a weekly basis, negatively affecting CDKs-complexes. Conversely, mitogens acting through Akt cause the phosphorylation and cytoplasmic localization of both p21 and p27, interfering with the cell-cycle progression [16, 34].

Pro-apoptotic and apoptotic proteins were investigated in this study to verify these results. Increased Bax and BcL-xL expression was detected in leiomyoma cells treated with E_2_ (100 nmol/L) and its combination with P_4_ (100 nmol/L) (Fig 7G and 7H). The trend towards a balance between Bax and BcL-xL was observed in both leiomyoma and myometrial adjacent cells in these conditions (Fig 7G–7J) and resulted from the relationship between the Bcl2 proteins family and p53 with consequent effects on cell cycle and cell death [36–38].

## Conclusion

A thorough characterization of primary leiomyoma and myometrial cells from uterine leiomyoma and myometrium adjacent tissues was conducted in this study. We used classic and well-established cell culture procedures for isolating and cultivating these cells. Thereby, these isolated primary leiomyoma and myometrial cells maintained the expression of smooth muscle and myoblast markers, retained the ability to respond to steroid hormones that trigger the MAPK signaling activation, and present an intermediate phenotype between that of myoblasts and myocytes.

The results from this study contribute to the evidence that no technique should be neglected to ensure the quality of primary cell cultures. Current cell culture models should be re-evaluated and improved in mimicking the in vivo environment according to the study’s goal in using the culture of primary cells to understand physiological mechanisms. Primary cell lines present the following limitations—they are not well characterized, have limited life span, show slow proliferation, and cannot be compared with other cell lines. Furthermore, primary cells derived from different patients can behave differently in culture conditions depending on the genetics and age of patients or individuals from whom the tissue was derived. Their characteristics change over time through passages; the cell population is not the same between passage 1 and 5 for example, which can skew experimental results. In addition, if primary cells are grown to very high confluency, the selection of subpopulations of cells adapted to outgrow slow growing cells might occur. The subculture of primary cells over an extended time leads to a continual selection of subpopulations of cells. This process might involve the selection of cells that are capable of proliferating on plastic surfaces, which are not the type of surface they attach inside the body. Nevertheless, these same cells fail to proliferate on soft substrates that approximate human tissues. Thus, working with the same cell population in the same experiment when using primary cells is imperative. Nevertheless, despite all these difficulties, work with primary culture mimic the diversity of responses observed in patients, and reveals the beauty of biology, that fibroblasts derived from different parts of the body may have different characteristics.

The last and most important note is that clinical trials have been conducted based on results obtained from the use of wrong cell lines; journals are starting to request researchers to validate their cell lines before publishing their data.

We suggest that the use of cell lines combined with primary cells would be preferable in most experiments, as long as they are properly handled, because in combination they could ensure consistency of results from an experiment to experiment.

This thorough characterization of primary leiomyoma and myometrial cells from uterine and myometrium adjacent tissues was fundamental for understanding the complex mechanisms underlying the pathogenesis of myometrium-derived diseases, notably leiomyomas.

## Supporting Information

S1 Fig Melting curve profile indicating the specific product. Melt curve (A) for GAPDH; (B) β2-microglobulin (endogenous control).
(DOCX)
