# Supplementary material for: Primary Human Uterine Leiomyoma Cell Culture Quality Control: Some Properties of Myometrial Cells Cultured under Serum Deprivation Conditions in the Presence of Ovarian Steroids
Source: PLoS One. 2016 Jul 8;11(7):e0158578. doi: 10.1371/journal.pone.0158578 (PMC4938619; doi:10.1371/journal.pone.0158578)
Supplement: S1 Fig — Melt curve (A) for GAPDH; (B) β2-microglobulin (endogenous control). (DOCX) [file pone.0158578.s001.docx]

**S1 Fig. Semi-quantitative RT-PCR**

Melt curve of endogenous control.

*
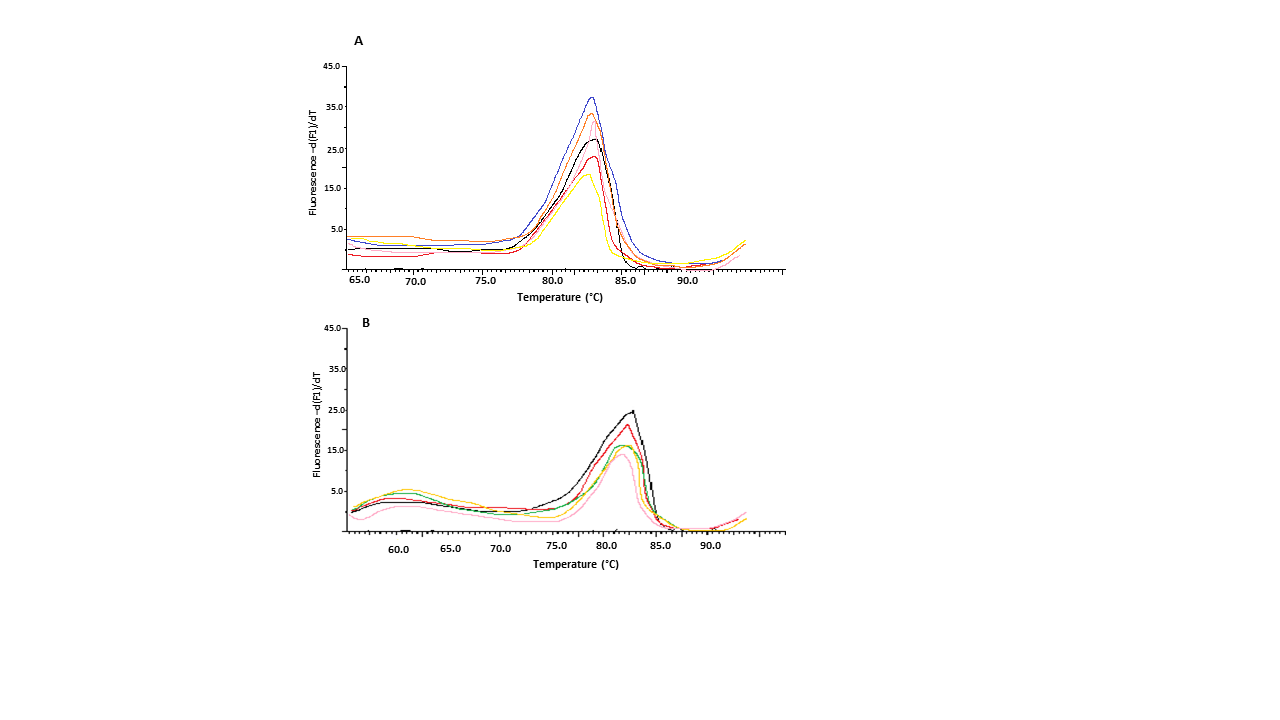
***S1 Fig.** Melting curve profile indicating the specific product. Melt curve (A) for GAPDH; (B) β2-microglobulin (endogenous control).
